# Echinacoside Induces Mitochondria-Mediated Pyroptosis through Raf/MEK/ERK Signaling in Non-Small Cell Lung Cancer Cells

**DOI:** 10.1155/2022/3351268

**Published:** 2022-05-06

**Authors:** Ye Shi, Hui Cao, Zhengcheng Liu, Lei Xi, Changqing Dong

**Affiliations:** ^1^Department of Thoracic Surgery, Nanjing Chest Hospital, Nanjing, Jiangsu, China; ^2^Department of Thoracic Surgery, The Affiliated Nanjing Brain Hospital of Nanjing Medical University, Nanjing, Jiangsu, China; ^3^Department of Thoracic Surgery, The Pulmonary Nodule Diagnosis and Treatment Research Center of Nanjing Medical University, Nanjing, Jiangsu, China

## Abstract

**Background:**

Various natural compounds are effective in cancer prevention and treatment with fewer side effects than conventional radiotherapy and chemotherapy. Considering the uncertainty of the antitumor mechanism of Echinacoside (Ech) and the fact that no study on Ech against non-small cell lung cancer (NSCLC) has been explored previously, this study inquired into the anti-NSCLC effect of Ech and explored its potential mechanisms.

**Methods:**

The IC_50_ to Ech of the NSCLC cells was calculated based on a series of cell viability assays. Different concentrations of Ech were used to treat the cells; the proliferation activity of the cells was evaluated using EdU staining. Mitochondrial membrane potential was detected by JC-1 staining. Levels of cytokines IL-1*β* and IL-18 were measured by ELISA. GSH and MDA levels were measured by microplate reader. Expression of cytochrome c, NLRP3, caspase-1, IL-1*β*, c-Myc, c-Fos, and Raf/MEK/ERK pathway proteins was evaluated by western blot. Meanwhile, we used xenograft, immunohistochemical staining, and H&E staining to evaluate the pharmacological effects of Ech in mice *in vivo*.

**Results:**

ECH inhibited the proliferation of NSCLC cells. Ech increased the expression of pyroptosis-related proteins. Besides, Ech perturbed the mitochondrial membrane potential with the release of mitochondrial cytochrome c, accompanied by increased oxidative stress. Ech inhibited the phosphorylation levels of Raf/MEK/ERK signaling pathway and subsequently reduced c-myc and c-fos protein expression. In addition, Ech effectively restrained the growth of tumors *in vivo*.

**Conclusions:**

Ech inhibited the Raf/MEK/ERK signaling. Impaired mitochondria activated inflammasome, which in turn led to the pyroptosis of NSCLC cells. These findings can provide some ideas on how to use pyroptosis to treat NSCLC.

## 1. Introduction

Lung cancer is one of the most common malignancies worldwide and the leading cause of cancer deaths. Non-small cell lung cancer (NSCLC) accounts for almost 80% of all lung cancers [1]. Despite improvements in early diagnosis and tremendous developments in platinum-based chemotherapy over the past decades, the prognosis and survival rates of NSCLC patients remain unsatisfactory [2]. In addition, its drug resistance, high recurrence rate, and high metastasis have gradually emerged [3]. Thus, the search for new highly effective and low-toxic anti-lung cancer drugs remains an extremely urgent task.

Natural products of plant origin have long been considered a source of anti-cancer agents and are being evaluated for several types of cancers [4–7]. Echinacoside (Ech) is one of the main phenylethanol glycosides isolated and purified from *Cistanche tubulosa*, which has been used as traditional Chinese herbal medicine with anti-senility and anti-fatigue effects [8–10]. Ech is composed of a phenyl propionic acid and a phenyl ethanol glycoside linked to a trisaccharide molecule. It is a hydrophilic polyphenol glycoside with intense scavenging activity of superoxide anion, hydroxyl radical, and lipid radical and can inhibit the self-oxidation of linoleic acid [11]. Ech contains caffeyl and hydroxy phenethyl molecules and exhibits various biological activities such as scavenging free radicals and antioxidant and anti-inflammatory effects [12, 13], considering the uncertainty of Ech in the mechanism of antitumor action and the fact that no studies on Ech against NSCLC have been reported so far. Therefore, this study will investigate the anti-NSCLC effect of Ech and explore its mechanism of action to some extent.

Pyroptosis is an inflammatory programmed cell death pathway, and the existing body of research suggests that pyroptosis was associated with inflammation responses in tumor cells. For instance, ophiopogonin B alleviates cisplatin resistance of lung cancer cells by inducing pyroptosis [14]. Hydrogen inhibits endometrial cancer growth via ROS/NLRP3/caspase-1-mediated pyroptotic pathway [15]. Polyphyllin VI induces caspase-1-mediated pyroptosis in NSCLC [16]. Therefore, triggering pyroptosis is considered a novel approach to treat cancers. Prior studies have noted the importance of the effect of mitochondria on pyroptosis. Mitochondria dysfunction has been shown to be implicated in the induction of pyroptosis [17–19]. Potential associations between the mitochondria and pyroptosis are indicated from previous studies. Of the tumor-related studies to date, few concerns about this aspect. Thus, the mitochondria-mediated pyroptosis is an issue that merits further research.

The reason why tumor cells could proliferate abnormally is closely related to their apoptosis defect and normal uncontrolled growth and differentiation. Recent studies have found that caspases, Bcl-2, Fos, and c-Myc gene families are widely involved in regulating apoptosis [20–23]. MAPK and other signaling pathways play principal roles in regulating cell growth and proliferation [24–27]. The aberrant activation of this pathway is inextricably linked to the generation of NSCLC and the evolution of nausea. The expression of Raf and its downstream genes MEK and ERK have been reported to be upregulated in NSCLC patient samples [28, 29]. Intracytoplasmic Raf is activated in response to stimulation by multiple potential oncogenic factors. On the one hand, activated Raf activates downstream MEK, which in turn phosphorylates ERK, after which the activated ERK enters the nucleus and alters its gene expression by phosphorylating related transcription factors, which in turn leads to abnormal cell proliferation. On the other hand, activated Raf can bind to Bcl-2 to form a dimer, and when it is transferred to mitochondria, Raf can separate it from Bcl-2 by phosphorylating BAD to increase the amount of free Bcl-2, thus inducing the activation of Bcl-2 anti-apoptotic function [30–32]. Herbal medicines are gaining popularity for the treatment of chronic and complex diseases as it works by hitting multiple molecular targets to modulate signaling pathways. An example of this is the study carried out by Huang [33] in which a biosynthetic ginsenoside mediated lung cancer by inhibiting Raf/MEK/ERK signaling pathway. Also, this is evident in the case of *Marsdenia tenacissima* extract suppressed lung cancer through ERK activation [34]. This efficacy was further exemplified in studies using Ech repressed pancreatic carcinoma cell growth by modulating MAPK activity [35], thus suggesting that Ech might have a positive therapeutic effect on tumors. Therefore, inhibition of Raf/MEK/ERK pathway activation is expected to be developed as a therapeutic pathway for NSCLC.

This study adequately confirmed that Ech could manifest anti-NSCLC effects in *in vitro* experiments by various experimental methods such as CCK-8, EdU, and ELISA. Subsequently, we found that Ech showed specific inhibition of the Raf/MEK/ERK signaling pathway. Finally, we used the NSCLC A549 xenograft tumor model to confirm *in vivo* that Ech could exert anti-NSCLC effects by inhibiting the activity of the Raf/MEK/ERK signaling pathway to mediate mitochondrial dysfunction and induce pyroptosis.

## 2. Materials and Methods

### 2.1. Cells and Chemicals

As previously described, cell culture was performed [36]. Human NSCLC cell lines A549 and H1299 cells were purchased from the CellBank (Shanghai, China) and cultured in DMEM medium (containing 10% fetal bovine serum-penicillin double-antibody solution). The cells were incubated in an incubator at 5% CO_2_ and 37 °C. The cells at the logarithmic growth stage were obtained at 2~3 d intervals. DMSO was used to make a stock solution of Ech (MedChemExpress, USA), which was diluted to a series of concentrations (25, 50 and 100 *μ*M) using growth medium [37]. Standard growth medium containing 0.1% DMSO was used as a control. To investigate the role of MAPK signaling in NSCLC cells of human origin, the inhibitor blocking component of the MAPK signaling, LM22B-10 (Abcam), was added to the cultures.

### 2.2. CCK-8 Viability Assay

A cell viability assay was performed to evaluate the effect of Ech on the NSCLC cells as described earlier [38]. The cells in healthy culture were digested with trypsin, 1200 r/min, centrifuged for 3 min. The cells were collected and counted. The cells were laid on 96-well plates with 3 × 10^3^ cells/well. The cells were treated with different concentrations of Ech for 24 h or 48 h, and 10 *μ*L CCK-8 (5 *μ*g/mL) solution was added 4 h before the cells were collected. Then, all the culture media were removed, and the cells were fully lysed, and the crystals were dissolved at room temperature for 10 min. The absorbance was measured by the enzyme-linked immunometric meter (450 nm wavelength).

### 2.3. EdU Staining Assay

EdU proliferation assay was performed, as previously described [39]. The cells were digested by trypsin, and the cells were counted by cell counter with 96-well plates (central plate, peripheral holes filled with blank medium) for 3 × 10^3^ cells. On the second day, Ech was added at different concentrations for 24 h, and EdU was added 4 h before the cells were collected. EdU was determined according to the recommended method of the instruction of Edu kit, and the corresponding inhibition rate was calculated.

### 2.4. Enzyme-Linked Immunosorbent Assay (ELISA)

Evaluation of the mitochondrial glutathione (GSH) level and the concentration of malondialdehyde (MDA), a lipid peroxidation product *in vitro* and *in vivo*, were measured by microplate reader according to the kit instructions as described previously [40].

### 2.5. Western Blotting

Western blot was performed as previously reported [41]. The cell lysate containing protease inhibitor was added for total protein extraction. Protein content was determined by BCA kit. The exact amount of protein sample (20 *μ*g) was extracted and denaturated at 100 °C for 5 min. Then, SDS-PAGE gel electrophoresis was performed for separation and transferred to PVDF membrane. 5% BSA was sealed at room temperature for 1~2h, the corresponding primary antibody (anti-NLRP3 [LS-C279941, 1 : 1000, LifeSpan], anti-caspase1 [LS-C211766, 1 : 1000, LifeSpan], anti-IL-1beta [LS-C154106, 1 : 1000, LifeSpan], anti-cytochrome c [LS-C317205, 1 : 1000, LifeSpan], anti-ERK1/2 [ab209321, 1 : 5000, Abcam], anti-p-ERK1/2 [ab201015, 1 : 1000, Abcam], anti-Raf [PA5-29333, 1 : 2000, Invitrogen], anti-p-Raf [44-504G, 1 : 1000, Invitrogen], anti-MEK1/2 [ab178876, 1 : 20000, Abcam], anti-p-MEK1/2 [ab278564, 1 : 1000, Abcam], anti-c-Myc [sc-40 AC, 1 : 500, Santa Cruz], anti-c-Fos [sc-7202, 1 : 500, Santa Cruz], and anti-beta-actin [ab20272, 1 : 5000, Abcam]) was added and incubated overnight at 4 °C. After cleaning, horseradish peroxidase-labeled secondary antibody was added and incubated at room temperature for 1 h. Finally, the luminescent solution was added and exposed to the gel imager, and the relative expression was calculated using Image J software. At least three independent experiments were repeated with *β*-actin as reference for sample loading.

### 2.6. Mitochondrial Membrane Potential (MMP) Assay

MMP assay was performed as described previously [42]. The cells in each group were inoculated on 6-well plates, incubated for 24 h, and added with JC-1 (Beyotime, Beijing, China) at room temperature to avoid light reaction for 40 min. The intensity of red and green fluorescence was measured and recorded by flow cytometry.

### 2.7. *In Vivo* Tumorigenicity

BALB/c female nude mice (5-6 weeks; Vitalriver, Beijing, China) weighing ~20 g were housed according to SPF class husbandry standards. The Ethics Committee of the Laboratory Animal Center of Nanjing Chest Hospital Affiliated to Southeast University approved the animal experiments. After the mice were acclimated to the experimental conditions, 10 BALB/c female nude mice were inoculated with A549 cells (5 × 10^6^ cells) in the right lower abdomen. The mice were then monitored for tumor size and randomly divided into control and Ech-treated groups (*n* = 5). The Ech-treated group was given saline containing 10 mg/kg Ech, the Ech dose was selected based on previous study [37], and the control group was given the same volume of saline [43]. The tumors were administered every three days, and the tumor size was measured simultaneously to plot the growth curve. 30 days later, the mice were executed by CO_2_. The tumors were removed separately, weighed and some tissues were fixed in 4% paraformaldehyde, the tumor diameter was measured by s caliper, and the rest were frozen at -80 °C for subsequent experiments.

### 2.8. Hematoxylin-Eosin (H&E) Staining

H&E staining was performed as described previously [44]. The tumor-bearing mice were weighed and killed, and the tumor tissues were peeled off. The 1 cm ×1 cm tissues were fixed in 10% formaldehyde solution and routinely embedded in paraffin wax for sectioning. H&E staining was used to observe the histopathological changes of the tumor under microscope.

### 2.9. Immunohistochemical (IHC) Staining

IHC was performed described previously [44]. Wax blocks containing tissue blocks were sectioned. The reaction was fulfilled for 30 min with a blocking solution containing 10% calf serum (Gicbo, Life Technologies, Carlsbad, CA). The primary antibodies (anti-Ki-67 [ab271811, 1 : 100, Abcam], anti-NLRP3 [ab214185, 1 : 100, Abcam], anti-caspase-1 [ab62698, 1 : 100, Abcam], and anti-IL-1*β* [ab283818, 1 : 500]) were added overnight at 4 °C, and PBS (Hyclone, Logan, USA) was used as a negative control instead of primary antibodies. The secondary antibodies were added dropwise with biotin-labeled IgG (ab205719, 1 : 1000, Abcam) and reacted at 37 °C for 1 h. Finally, DAB (Dako, Denmark) was used for color development and hematoxylin contrast staining and sealed with gelatin. The scoring criteria for immunohistochemistry were done by the physicians of the pathology department of our hospital.

### 2.10. Statistical Analysis

All results were analyzed using GraphPad Prism 8.0 (GraphPad Software, Inc.). One-way analysis of variance (ANOVA) followed by the post hoc Dunnett's test was used for the comparison among multiple groups. Student's *t*-test was used to calculate the differences among the groups, and the *p* value was less than 0.05, which was statistically significant.

## 3. Results

### 3.1. Ech Restrained the Activity of NSCLC Cells

**Figure 1:**
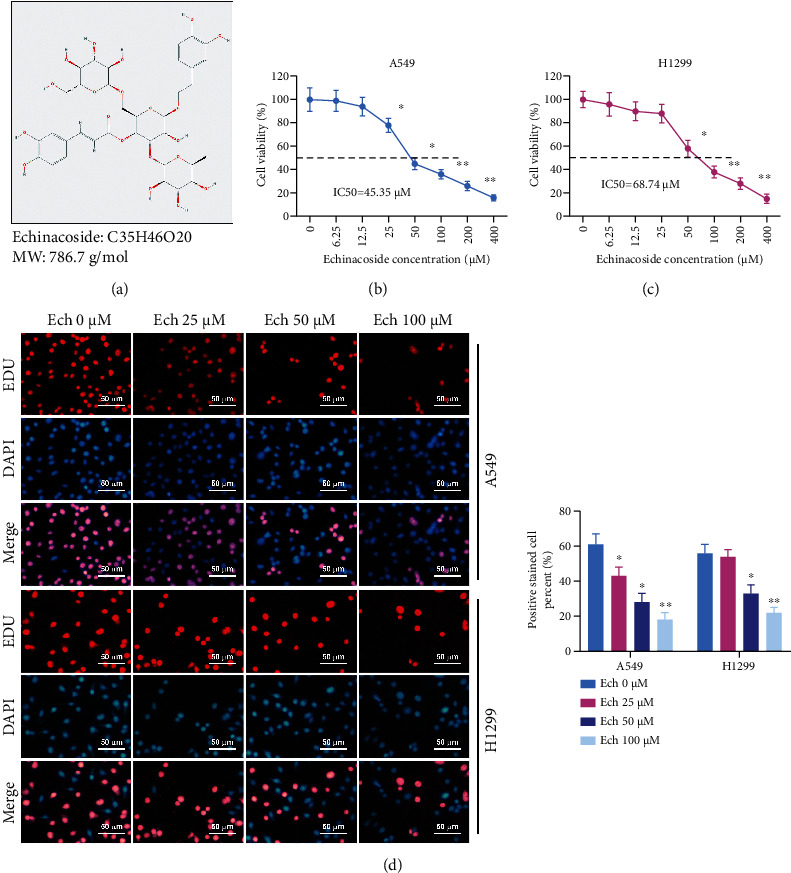
Ech restrained the activity of A549 and H1299 cells. (a) Molecular framework of Ech. (b, c) Cell viabilities of A549 and H1299 cells measured by the CCK-8 method under Ech treatments were used to calculate the IC_50_ values. (d) The proliferation suppression impact of Ech in A549 and H1299 cells was further verified by EdU assay and relative EdU-positive ratio, respectively. Bar = 50 *μ*m. Data were represented as mean ± SD. ∗*P* < 0.05 and ∗∗*P* < 0.01.

Ech, the major component of the stem of *Cistanche tubulosa*, is a phenylethanol glycoside with the structure shown in Figure 1(a). Cell survival was measured by CCK-8 methods after Ech treatment of two NSCLC cell lines for 24 h. ECH significantly inhibited the growth of A549 and H1299 cell lines. The IC_50_ values were 45.35 *μ*M and 68.74 *μ*M, respectively (Figures 1(b) and 1(c)). Therefore, concentrations of 25-100 *μ*M were chosen for subsequent experiments. The Ech in the doses of 25 *μ*M, 50 *μ*M, and 100 *μ*M could effectively inhibit cell viability, with the high dose group having the most pronounced effect on cell viability (Figure 1(d)). Initially, it reflected that Ech could inhibit NSCLC cell tumorigenesis *in vitro*.

### 3.2. Ech Induced Pyroptosis in NSCLC Cells

**Figure 2:**
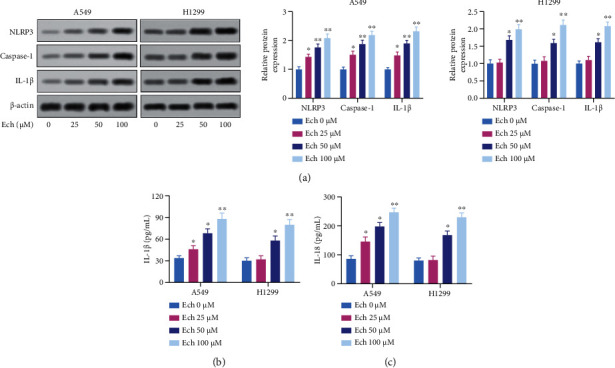
Ech induced pyroptosis in NSCLC cells. (a) Representative western blot showing protein levels of NLRP3, caspase-1, and IL-1*β*. (b) Supernatants were analyzed for IL-1*β* by ELISA. (c) The contents of IL-18 were detected by ELISA. Data were represented as mean ± SD. ∗*P* < 0.05 and ∗∗*P* < 0.01.

Given NLRP3 inflammasome activation is closely associated with pyroptosis [45], we further investigated whether pyroptosis occurred in the cells treated with Ech. First, in NSCLC cells after treating with gradient concentrations of Ech, we measured the levels of pyroptosis-related proteins NLRP3, caspase-1, and IL-1*β*. In A549 and H1299 cells, the above proteins were increased under Ech treatment to some extent (Figure 2(a)). Similarly, both IL-1*β* and IL-18 tended to increase under Ech treatment (Figure 2(b)). These results suggested that Ech might induce NSCLC cells to undergo pyroptosis.

### 3.3. Ech Led to Mitochondrial Dysfunction in NSCLC Cells

**Figure 3:**
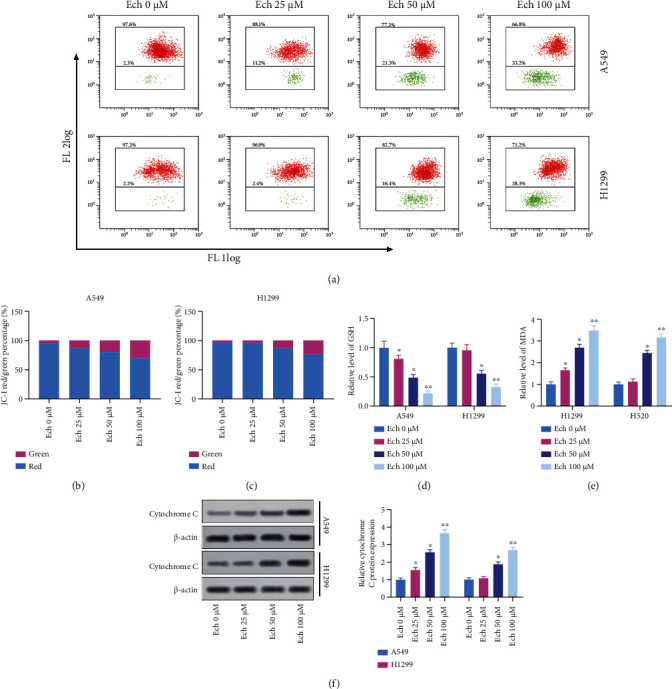
Ech led to mitochondrial dysfunction in NSCLC cells. (a) The mitochondrial membrane potential was detected by JC-1 staining. (b, c) Evaluation of mitochondrial membrane potential. (d, e) Oxidative stress markers (MDA and GSH) were assessed. (f) Cytochrome c release during Ech treatment was detected by western blot. Data were represented as mean ± SD. ∗*P* < 0.05 and ∗∗*P* < 0.01.

The disruption of the mitochondrial transmembrane potential often accompanies the process of programmed cell death [46]. After the different concentrations of Ech (0, 25, 50, and 100 *μ*M) were applied for 12 h, the mitochondrial membrane potential of the cells all showed different levels of decrease, especially at high concentrations of Ech; the decrease was abnormally significant (Figure 3(a)–3(c)). Meanwhile, oxidative stress was assessed by measurements of NSCLC cells GSH and MDA; decreased levels of GSH and increased levels of MDA were observed (Figure 3(d)). Similarly, Ech led to cytochrome c releasing into the cytoplasm (Figure 3(f)). These results demonstrated that Ech could induce early apoptosis of the cells and thus promote the death of tumor cells.

### 3.4. Ech Restrained the Malignant Phenotypes of NSCLC Cells by Inhibiting Raf/MEK/ERK Signaling Pathway Activation

**Figure 4:**
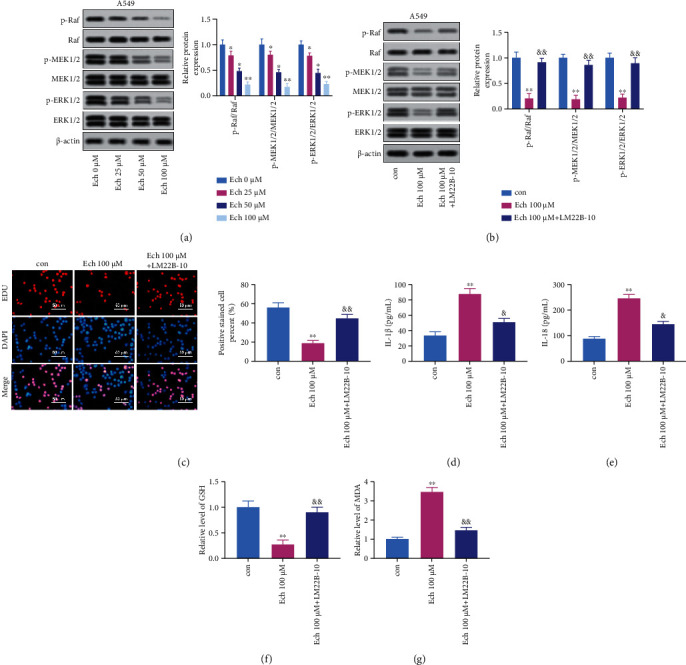
Ech restrained the malignant phenotypes of NSCLC cells by inhibiting Raf/MEK/ERK signaling pathway activation. (a) The expression of Raf/MEK/ERK signaling pathway proteins, c-Myc, and c-Fos in A549 cells was detected by western blotting. (b) The phosphorylation of the Raf/MEK/ERK signaling, c-Myc, and c-Fos was measured by western blotting in A549 cells treated by Ech with or without LM22B-10. (c) EdU staining of A549 cells and EdU-positive cell proportion. (d, e) Levels of IL-1*β* and IL-18 in cell culture supernatants were assessed by ELISA. (f, g) Ech decreased GSH and increased MDA. Bar = 50 *μ*m. Data were represented as mean ± SD and represent three replicated experiments. ∗*P* < 0.05 versus Ech 0 *μ*M; ∗∗*P* < 0.01 versus Ech 0 *μ*M; &, *P* < 0.05 versus Ech 100 *μ*M; &&, *P* < 0.01 versus Ech 100 *μ*M.

The Raf/MEK/ERK signaling pathway is one of the MAPK pathways, and its aberrant activation associates with various tumorigenic diseases [47]. Therefore, we speculate whether Ech-induced NSCLC cell pyroptosis is associated with the inhibition of this signaling pathway. Ech treatment activated Raf/MEK/ERK pathway and promoted apoptosis. Total protein quantity of Raf, MEK1/2 and ERK1/2 did not change significantly, but their phosphorylated forms were significantly downregulated with the increase of Ech concentration. Furthermore, the expression of c-Myc and c-Fos, the downstream proteins of this signaling pathway, decreased with increasing concentrations of Ech (Figure 4(a)). To clarify that Ech induces pyroptosis by arresting the Raf/MEK/ERK signaling pathway, which ultimately leads to a decrease in p-ERK1/2 protein expression, we conducted further experiments by adding a p-ERK agonist (LM22B-10). The results showed that the agonist greatly increased the phosphorylation level of ERK1/2 protein, likewise the expression of c-Myc and c-Fos (Figure 4(b)). To determine the implications of enhanced ERK activation in cell activity, we performed the EdU uptake assay. Interestingly, the ERK agonists LM22B-10 increased the EdU-positive proportion (Figure 4(c)). Subsequently, the cells were restimulated with LM22B-10. In contrast with Ech treatment alone, LM22B-10 reduced the secretion of IL-1*β* and IL-18 (Figures 4(d) and 4(e)). Similarly, the ERK agonists LM22B-10 could increase GSH levels while decrease MDA levels (Figures 4(f) and 4(g)). These data implied that Ech induces pyroptosis by decreasing the phosphorylation levels of Raf/MEK/ERK signaling.

### 3.5. Ech Frustrated the Growth of NSCLC Cells *In Vivo*

**Figure 5:**
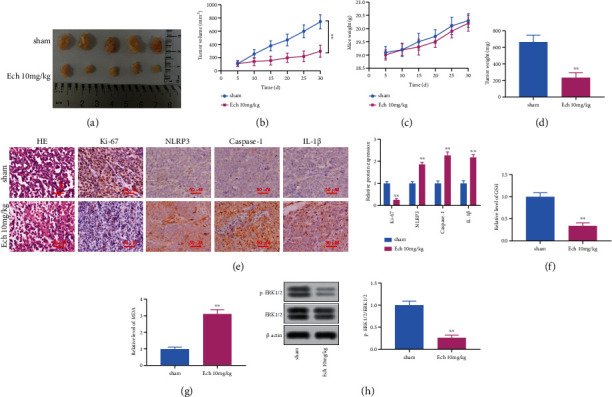
Ech frustrated the growth of NSCLC cells *in vivo*. (a) Representative tumorigenic images. (b) Statistical chart of tumor volume. (c) Average weight of mice (calculated from group weight divided by number of mice weighted) taken over a course of 30 days. D Statistical chart of tumor weights. E Results of H&E staining and immunohistochemistry of tumor tissues (Bar =50 *μ*m). Determination of GSH (f) and MDA (g) levels in the tumor tissues. (h) Total lysates were harvested for the assay of ERK1/2 and phosphorylated ERK1/2 protein levels using western blot analysis. Data were represented as mean ± SD. ∗∗*P* < 0.01.

Previous studies have shown that many drugs only *in vitro* experiments can produce good results, but *in vivo* experiments cannot work effectively, which limits the use of drugs. Therefore, we are eager to confirm whether Ech could produce an effective antitumor effect *in vivo*. Therefore, we constructed the classical A549 nude mice model of tumor formation and used Ech intervention treatment to observe its efficacy. The tumors were weighed and photographed (Figure 5(a)). Compared to the control group, Ech group remarkably reduced the size (Figure 5(b)) and weight (Figures 5(c) and 5(d)) of the tumors, and by plotting the tumor growth curve, the tumor growth was significantly slowed down. Furthermore, the inhibition of Ech on the tumor *in vivo* was proved by morphological experiment. The nucleus density of the Ech group decreased, and the vacuolation of the cytosolic staining increased after H&E staining. Ki-67, NLRP3, caspase-1, and IL-1*β* expression were observed by IHC. The levels of proteins above were considerably decreased after the treatment with Ech (Figure 5(e)). Turning now to the experimental evidence on oxidative stress, the GSH serum level decreased, while the serum levels of MDA increased in the model mice (Figures 5(f) and 5(g)). Ech significantly reduced p-ERK1/2 levels in tumor tissues, while p-ERK1/2 levels in the model control group were not significantly altered (Figure 5(h)), suggesting that the *in vivo* antitumor mechanism of Ech was with respect to the inhibition of Raf/MEK/ERK signaling pathway.

## 4. Discussion

Previous studies have shown that conventional radiotherapy and chemotherapy are effective in controlling cancer progression. However, it is often accompanied by severe side effects. Thus, the search for new therapeutic agents is particularly important. A growing number of studies have shown that various natural compounds are effective in cancer prevention and treatment with fewer side effects. In the current study, we confirmed that Ech effectively arrests the growth of NSCLC cells both *in vitro* and *in vivo*. The putative mechanism of the effects is to mediate mitochondrial dysfunction and subsequent induction of pyroptosis by reducing the activation of the Raf/MEK/ERK signaling pathway.

There is an increasing number of studies on the antitumor effects of Ech. Ech treatment of the hepatocellular carcinoma cell line HepG2 showed a concentration-dependent decrease in the expression of phosphorylated AKT and an increase in the expression of the cell cycle inhibitor p21 and the pro-apoptotic protein Bax [48]. The expression level of TREM2 was significantly decreased when Ech was treated with HepG2 cells or DEN-induced hepatocellular carcinoma mouse model, and the inhibitory effect of Ech was reduced by overexpression of TREM2 [48]. It was concluded that the mechanism of Ech inhibition of hepatocarcinogenesis was through downregulation of TREM2 and inhibition of PI3K/AKT signaling pathway. In other studies, Ech has been shown to inhibit the proliferation of prostate cancer cells [49]. Molecular biological assays revealed that Ech treatment of colorectal cancer cells increased caspase-3 activation and upregulated the cell cycle inhibitor protein p21. The potential mechanism of effect was found to be through oxidative DNA damage in tumor cells [50]. In addition, Ech treatment-induced reactive oxygen species production and altered mitochondrial membrane potential, which inhibited pancreatic cancer cell proliferation and promoted apoptosis, and its proposed mechanism of effect was through the regulation of MAPK signaling pathway [35], suggesting that Ech functioning as an adjuvant drug in treating tumor. In the present study, Ech treatment considerably inhibited NSCLC cell proliferation. Mechanistically, Ech contributed to mitochondrial dysfunction by inhibiting the phosphorylation levels of the Raf/MEK/ERK signaling pathway, which in turn induces pyroptosis.

Generally, the development of malignancy is attributed to multiple factors, including the activity of proto-oncogenes and oncogenes, the immune microenvironment, and the inflammatory microenvironment [51]. In contrast, the proinflammatory nature of pyroptosis makes it relevant to the pathogenesis of various chronic inflammatory diseases. Thus, the inflammatory microenvironment formed by the release of large amounts of inflammatory factors during the pyroptosis process has the potential to promote tumor development. It has been found that NLRP3 and its mediated production of IL-1*β* could promote the development of inflammatory bowel disease, which, eventually, might develop into gastrointestinal malignancies, thus suggesting that pyroptosis actions an essential part in this process. In addition, sorafenib played an anticancer role by inducing pyroptosis in macrophages, followed by triggering and enhancing the immune response of NK cells against HCC cells [52]. Downregulation of the expression of inflammasomes mediating pyroptosis led to the cell proliferation, while downregulation of GSDMD significantly facilitated the cell proliferation of gastric cancer [53]. In therapeutic studies in other malignancies, pyroptosis could also play a role in inhibiting the proliferation and metastasis of malignant tumor. The proinflammatory nature of pyroptosis might contribute to the development of malignant tumors, but its effect on malignant tumors has been confirmed by studies. Several was found in the literature on the question of the effect of Ech on oxidative stress and inflammation; it has shown that Ech diminished serum markers of oxidative stress and inflammation in diet/streptozotocin-induced diabetic rats and also retinal ganglion cells [54, 55]. The NLRP3/caspase-1 pathway is a critically involved pathway in the inflammatory response. In the present study, IL-1*β* produced by pyroptosis mediated the inactivation of the downstream Raf/MEK/ERK signaling pathway and finally inhibited the proliferation of NSCLC, thus illustrating the role of pyroptosis in NSCLC. However, based on the identified inflammasomes, the caspase family proteins, GSDM family proteins, and related signaling pathways, we are searching for effective therapeutic targets and developing targeted drugs. Ech upregulated the expression of NLRP3 and caspase-1 in nude mice NSCLC transplant tumor tissues and inhibited the growth of nude mice NSCLC transplant tumors. This could lead to a breakthrough point in the treatment of malignant tumors using pyroptosis.

MAPK is a class of kinases widely present in mammalian cells that can participate in manifold processes for instance cell extension, development, differentiation, apoptosis, and intercellular functional synchronization by mediating the conversion of extracellular molecules by growth factors, hormones, and other cytokines [56]. Previous studies have consistently concluded that MAPK cascade reactions, and their upstream promoters are abnormally regulated to induce the development of many human tumors and other diseases [57, 58]. Among the multiple proteins of the MAPK cascade, ERK1/2 is the most common. They action a crucial part in Raf/MEK/ERK signaling pathway controlling cell growth and differentiation, which are important members of the protein kinase cascade that transmits extracellular growth and neurotrophic cells to the nucleus [59]. In contrast, ERK1/2 has numerous downstream substrates, including the oncogene Bcl-2. It has been found in glioma that increased Ras protein activity activates the downstream Raf/MEK/ERK signaling pathway, leading to upregulation of oncogene Bcl-2 expression and ultimately immortalizing tumor cells [60]. In the present study, the highest activity of the Raf/MEK/ERK signaling pathway was observed in the group without Ech treatment, revealing that the effect of Ech on NSCLC cells is associated with the inhibition of Raf/MEK/ERK signaling pathway activity. Furthermore, ERK1/2 agonist induced the downregulation of c-Myc and c-Fos expression, indicating that the ERK1/2 pathway has regulatory effects on the expression of various proliferation genes in NSCLC cells, which is consistent with previous perceptions about genes downstream of the ERK1/2 pathway. The downregulation of c-Myc and c-Fos expression in NSCLC cells after Ech treatment was consistent with the inhibition of NSCLC cell proliferation and p-ERK1/2 expression by Ech, which further confirmed the regulatory effect of Ech on ERK1/2 pathway. LM22B-10 (C_27_H_33_ClN_2_O_4_) is a typical ERK agonist, capable of precisely inducing ERK activation *in vitro* and *in vivo*, and is more commonly used in studies targeting the signaling pathways ERK and TrkB. It has been shown in several studies to activate ERK and thus affect the biological behavior of cells [61, 62]. It has been demonstrated that sevoflurane could play a role in inhibiting the proliferation and invasion of colon cancer cells by downregulating the ERK signaling pathway, and LM22B-10 promoted the proliferation and invasion of cancer cells and inhibited apoptosis and autophagy by activating the ERK signaling pathway [63]. In this study, we added ERK agonist LM22B-10 on top of the drug treatment to further investigate whether this signaling pathway of Raf/MEK/ERK plays a role in this model. LM22B-10 partially restored the proliferation-inhibiting, inflammation-inducing effects of Ech on cancer cells. It indicated that Ech affecting the biological behavior of NSCLC cells might be related to the MAPK/ERK signaling pathway.

The mechanism of effects of Chinese medicine in the treatment of NSCLC is complex and involves multiple pathways. The regulation of intracellular molecular signaling is one of the common aspects. However, there are multiple signaling pathways in cells. They do not function individually but interact to form a complex signaling network, which systematically regulates the body as a whole to exert antitumor effects. Therefore, it is impossible to elucidate the mechanism of function systematically and holistically. Future research should explore the multifaceted and multisystemic regulatory mechanisms of the antitumor effects of traditional Chinese medicine in more meticulous detail and further investigate its antitumor mechanisms in a more accurate way.

## 5. Conclusion

In conclusion, Ech could effectively reduce the mitochondrial membrane potential of NSCLC cells and promote the occurrence of pyroptosis. Moreover, the mechanism by which Ech promotes pyroptosis in NSCLC cells consists in its inhibition of phosphorylated proteins p-Raf, p-MEK1/2, and p-ERK1/2 in the Raf/MEK/ERK signaling pathway. Certainly, Ech induces pyroptosis by decreasing the phosphorylation level of ERK1/2 protein.

## Data Availability

Analytical data generated during this investigation are available upon justified request to the corresponding author.

## Abbreviations

NSCLC: Non-small cell lung cancer
Ech: Echinacoside
GSH: Total glutathione
MDA: Malondialdehyde
ERK1/2: Extracellular signal-regulated kinase 1/2
CCK-8: Cell counting kit-8
DMEM: Dulbecco's modified eagle's medium
FBS: Fetal bovine serum
BSA: Bovine albumin
DAB: Diaminobenzidine
DMSO: Dimethyl sulfoxide
PVDF: Polyvinylidene fluoride
PAGE: Polyacrylamide gel electrophoresis
PBS: Phosphate buffered saline
NLRP3: NLR family pyrin domain containing 3
IL-1*β*: Interleukin 1*β*
IL-18: Interleukin 18
EdU: 5-Ethynyl-2'-deoxyuridine
ELISA: Enzyme-linked immunosorbent assay
IHC: Immunohistochemistry
H&E: Hematoxylin-eosin
MAPK: Mitogen activated protein kinase.
